# Chloroquine and Its Derivatives Exacerbate B19V-Associated Anemia by Promoting Viral Replication

**DOI:** 10.1371/journal.pntd.0000669

**Published:** 2010-04-27

**Authors:** Claudia Bönsch, Christoph Kempf, Ivo Mueller, Laurens Manning, Moses Laman, Timothy M. E. Davis, Carlos Ros

**Affiliations:** 1 Department of Chemistry and Biochemistry, University of Bern, Bern, Switzerland; 2 CSL Behring, Bern, Switzerland; 3 Vector Borne Disease Unit, Papua New Guinea Institute of Medical Research, Goroka, Papua New Guinea; 4 School of Medicine and Pharmacology, University of Western Australia, Crawley, Western Australia, Australia; Christian Medical College, India

## Abstract

**Background:**

An unexpectedly high seroprevalence and pathogenic potential of human parvovirus B19 (B19V) have been observed in certain malaria-endemic countries in parallel with local use of chloroquine (CQ) as first-line treatment for malaria. The aims of this study were to assess the effect of CQ and other common antimalarial drugs on B19V infection *in vitro* and the possible epidemiological consequences for children from Papua New Guinea (PNG).

**Methodology/Principal Findings:**

Viral RNA, DNA and proteins were analyzed in different cell types following infection with B19V in the presence of a range of antimalarial drugs. Relationships between B19V infection status, prior 4-aminoquinoline use and anemia were assessed in 200 PNG children <10 years of age participating in a case-control study of severe infections. In CQ-treated cells, the synthesis of viral RNA, DNA and proteins was significantly higher and occurred earlier than in control cells. CQ facilitates B19V infection by minimizing intracellular degradation of incoming particles. Only amodiaquine amongst other antimalarial drugs had a similar effect. B19V IgM seropositivity was more frequent in 111 children with severe anemia (hemoglobin <50 g/L) than in 89 healthy controls (15.3% vs 3.4%; *P* = 0.008). In children who were either B19V IgM or PCR positive, 4-aminoquinoline use was associated with a significantly lower admission hemoglobin concentration.

**Conclusions/Significance:**

Our data strongly suggest that 4-aminoquinoline drugs and their metabolites exacerbate B19V-associated anemia by promoting B19V replication. Consideration should be given for choosing a non-4-aminoquinoline drug to partner artemisinin compounds in combination antimalarial therapy.

## Author Summary

Human parvovirus B19 (B19V) is typically associated with a childhood febrile illness known as erythema infectiosum. The infection usually resolves without consequence in healthy individuals. However, in patients with immunologic and/or hematologic disorders, B19V can cause a significant pathology. The virus infects and kills red cell precursors but anemia rarely supervenes unless there is pre-existing anemia such as in children living in malaria-endemic regions. The link between B19V infection and severe anemia has, however, only been confirmed in certain malaria-endemic countries in parallel with chloroquine (CQ) usage. This raises the possibility that CQ may increase the risk of severe anemia by promoting B19V infection. To test this hypothesis, we examined the direct effect of CQ and other commonly used antimalarial drugs on B19V infection in cultured cell lines. Additionally, we examined the correlation between B19V infection, hemoglobin levels and use of CQ in children from Papua New Guinea hospitalized with severe anemia. The results suggest strongly that CQ and its derivatives aggravate B19V-associated anemia by promoting B19V replication. Hence, careful consideration should be given in choosing the drug partnering artemisinin compounds in combination antimalarial therapy in order to minimize contribution of B19V to severe anemia.

## Introduction

Human parvovirus B19 (B19V) is a nonenveloped icosahedral virus with a single-stranded DNA genome which has been classified within the *Erythrovirus* genus of the *Parvoviridae* family. The virus is readily transmitted via the respiratory route and has a worldwide distribution. Seroprevalence increases with age and 50%–80% of adults have detectable B19-specific antibody. Since its discovery in 1975 [1], B19V has been associated with an expanding range of clinical disorders that reflect the patient's immunologic and hematologic status. In healthy individuals, B19V typically causes a mild childhood febrile illness known as erythema infectiosum or fifth disease. More severe manifestations of B19V infection are arthropathies, aplastic anemia, hydrops fetalis and fetal death [2].

Viremia occurs during the first week of infection. The virus has a predilection for bone marrow erythroid progenitor cells. At the height of viremia, there is an abrupt fall in the reticulocyte count and anemia can supervene. Although this is rarely apparent in healthy patients, it can have serious clinical consequences where there is pre-existing anemia [2]. A case in point is malaria. B19V co-infection has been considered a significant risk for severe anemia in children living in malaria-endemic regions [3]. Studies examining the inter-relationship between malaria, B19V infection and anemia have, however, produced inconsistent results. In a retrospective study from Papua New Guinea (PNG) [4], 60% of children <2 years of age and 90% of 6 year-olds were B19V seropositive. B19V infection was significantly associated with severe anemia even in the absence of risk factors including malaria. Similar results were obtained in children from the Republic of Niger [5]. However, studies from Malawi and Kenya did not show evidence that B19V infection contributes to anemia in children and the seroprevalence of B19V was relatively low [6], [7].

At the time the studies were performed in PNG [4] and Niger [5], chloroquine (CQ) was used as first-line treatment for malaria in these countries. In addition, a serosurvey performed in Eritrea at a time when CQ was the first-line agent also revealed an unusually high B19V seroprevalence [8]. By contrast, CQ had been discontinued in Malawi because of resistance of local strains of *Plasmodium falciparum* and its use was declining in Kenya when the B19V seroprevalence studies were conducted [6], [7]. Although CQ has, in addition to antimalarial efficacy, broad antiviral activity [9], [10], it also enhances Semliki Forest virus (SFV) and encephalomyocarditis virus (EMCV) infection in mice [11], and Epstein-Barr virus (EBV) expression [12]. The latter effect is thought to play a role in the higher incidence of EBV-induced Burkitt's lymphoma in malaria-endemic areas where CQ is in common use [13].

We hypothesize, therefore, that geo-epidemiological differences in B19V seroprevalence and pathogenic potential result from CQ-associated enhanced replication. To test this hypothesis, we examined the effect of CQ and other commonly-used antimalarial drugs on B19V replication in three different cultured cell lines. In addition, we examined the relationship between B19V infection and use of 4-aminoquinoline drugs in a sample of children from PNG who were hospitalized with severe anemia. The results provide evidence that CQ and AQ aggravate B19V-associated anemia by promoting B19V replication.

## Methods

### Ethics statement

All patients were participants in a prospective observational and genetic study of severe pediatric infections (http://www.malariagen.net/home/). Written informed consent was obtained from each parent/guardian. Ethical approval for the study was obtained from both the PNG Institute of Medical Research Institutional Review Board and the Medical Research Advisory Committee of the PNG Department of Health. The study was conducted in accordance with the Helsinki Declaration.

### Cells and viruses

A B19V-infected plasma sample was obtained from our donation center (Genotype 1; CSL Behring AG, Charlotte, NC) and was concentrated by ultracentrifugation through 20% (w/v) sucrose. UT7/Epo cells were cultured in RPMI, 10% FCS and 2 U/ml of recombinant human erythropoietin (Epo; Janssen-Cilag, Midrand, South Africa). HepG2 cells were cultured in MEM supplemented with 10% FCS. Bone marrow mononuclear cells (BMMCs) were obtained as frozen stocks from Stemcell Technologies (Vancouver, BC, Canada) and were cultured in IMDM, 10% FCS and 2 U/ml of Epo. All cells were incubated at 37°C and in an atmosphere of 7.5% CO_2_.

### Drugs

All drugs were purchased from Sigma (St. Louis, Miss). Chloroquine diphosphate (CQ), primaquine diphosphate (PQ) and amodiaquine dihydrochloride dihydrate (AQ) were dissolved in water, piperaquine (PPQ) in 5% lactic acid, mefloquine hydrochloride (MQ) in DMSO, lumefantrine (LFT) in dimethylformamide and artesunate (AT) and pyrimethamine (PM) in ethanol. The final drug concentration ranges used in the B19V infectivity assay were 5–100 µM for PQ and LFT, AQ and PM, 0.05–20 µM for MQ, 2.5–100 µM for AT, 1–100 µM for PPQ and 0.05–100 µM for CQ. The highest concentration of the drugs did not exceed 0.2% of total culture volume.

### Infectivity assay

B19V infectivity was assessed in two different cell lines, namely megakaryoblastic leukemia UT7/Epo cells and the human hepatocellular liver carcinoma cell line HepG2, and in primary bone marrow mononuclear cells (BMMCs). UT7/Epo cells are the most susceptible cell line to B19V infection [14]. Although viral RNA, DNA and proteins can be detected in B19V-infected UT7/Epo cells, viral replication is restricted to a level that does not normally allow production of virus progeny. The HepG2 cell line was chosen because it allows virus binding and probably internalization, but it is non-permissive for B19V infection [15]. BMMCs have been shown to support B19V infection, although only a minor subset of these cells is permissive for B19V [16].

UT7/Epo, HepG2 and bone marrow mononuclear cells (BMMCs) (3×10^5^) were infected with 20,000 DNA-containing B19V particles per cell (5,000 for BMMCs), corresponding to an MOI of approximately 20 (5 for BMMCs) in the presence of the pre-determined concentrations of the selected drugs. For viral RNA and DNA analysis, cells were collected at different post-infection times as indicated in the figure legends. Total poly (A)^+^ mRNA was isolated and viral NS1 mRNA quantified as previously described [17]. Total DNA was extracted and viral DNA was quantified using established methods [17].

### Analysis of viral protein expression

**Figure 1:**
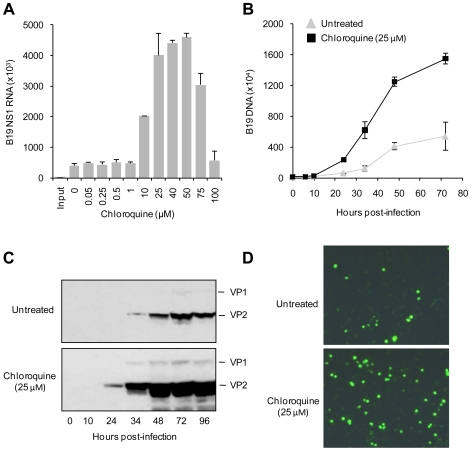
Enhancement of B19V transcription, replication and protein expression by CQ in UT7/Epo cells. Panel A shows the quantification of viral RNA synthesis 24 h after infection with B19V in the presence of increasing CQ concentrations. The results are the average of three independent experiments. SD bars are shown. Panel B shows the quantification of viral DNA replication at increasing post-infection times in untreated and CQ-treated (25 µM) cells. Values shown represent the average of two independent experiments. SD bars are shown. Panel C shows the kinetics of B19V structural protein expression in untreated and CQ-treated (25 µM) cells. Panel D shows the detection of viral protein expression by immunofluorescence in untreated and CQ-treated (25 µM) cells.

UT7/Epo cells were infected as specified above in the presence of 0 or 25 µM CQ. At increasing post-infection times (see figure 1C), cells were lysed in protein loading buffer and total proteins were resolved by sodium dodecyl sulfate (SDS)-10% polyacrylamide gel electrophoresis (PAGE). After transfer to a PVDF membrane, the blot was probed with a mouse antibody against B19V structural proteins (1∶2,000 dilution; US Biologicals, Swampscott, MA), followed by a horseradish peroxidase-conjugated secondary antibody (1∶20,000 dilution). The viral structural proteins were visualized with a chemiluminescence system (Pierce, Rockford, IL). Additionally, viral protein expression was examined by immunofluorescence, as previously described [17].

### Analysis of B19V DNA stability during intracellular trafficking

UT7/Epo cells (3×10^5^) were infected with 20,000 viral particles per cell (MOI 20) at 4°C for 2 h. The cells were washed 8 times with PBS to remove unbound virus and incubated at 37°C in the presence or absence of antimalarials. At increasing post-internalization times from 1 to 7 h, the cells were washed 2 times with PBS and the amount of intact viral DNA was quantified as specified above.

### Clinical study

We studied 111 children <10 years of age with severe anemia (hemoglobin <50 g/L) and 89 community-based age and sex-matched healthy control children with a hemoglobin >100 g/L. Those with severe anemia represented a subset (15.9%) of all 697 children admitted to Modilon Hospital, Madang Province on the north coast of PNG with any severe illness during the period of study. Modilon Hospital is a referral hospital and the only provincial facility able to manage severely ill children. All such children were given treatment as recommended under PNG national treatment guidelines including intramuscular artemether for malaria infection. The healthy controls were recruited from the same villages as the patients and were slide-negative for malaria.

As well as a hemoglobin concentration (HaemoCue®, Angelholm, Sweden) at presentation, plasma was assayed for B19V IgM by EIA kit (Biotrin International) and, in those with severe anemia, for viral DNA using two specific oligonucleotide primers [4]. We did both tests because viremia starts to decline once specific IgM is produced around day 9 after inoculation, while virus-induced marrow suppression can last for another 2–3 weeks [4]. Thus, although the simultaneous detection of B19V IgM and DNA is strongly indicative of acute infection, we did not want to exclude children with evidence of recent but resolving infection as a contributor to severe anemia. In those who were B19V IgM or PCR positive, plasma was assayed for chloroquine and amodiaquine and their respective active desethyl metabolites using a validated high performance liquid chromatography assay [18]. The assay had a limit of quantitation of 1 µg/L for each analyte.

## Results

### Effect of CQ on B19V replication in UT7/Epo cells

CQ increased the production of B19V NS1 gene transcription after 24 h incubation. At CQ concentrations ranging from 10 to 75 µM, NS1 RNA increased up to 1,170% (Figure 1A). Similarly, kinetic studies in the presence of 25 µM of CQ showed that viral DNA synthesis was more rapid and extensive than in untreated cells (Figure 1B). The expression of structural viral proteins in extracts of infected UT7/Epo cells was also increased in the presence of 25 µM CQ (Figure 1C). Viral protein expression was detectable by 34 h in untreated cells and by 24 h in CQ-treated cells. Immunofluorescence experiments showed, that in the presence of CQ a larger number of cells were infected by B19V (Fig. 1D).

### Effect of CQ on B19V replication in HepG2 cells

**Figure 2:**
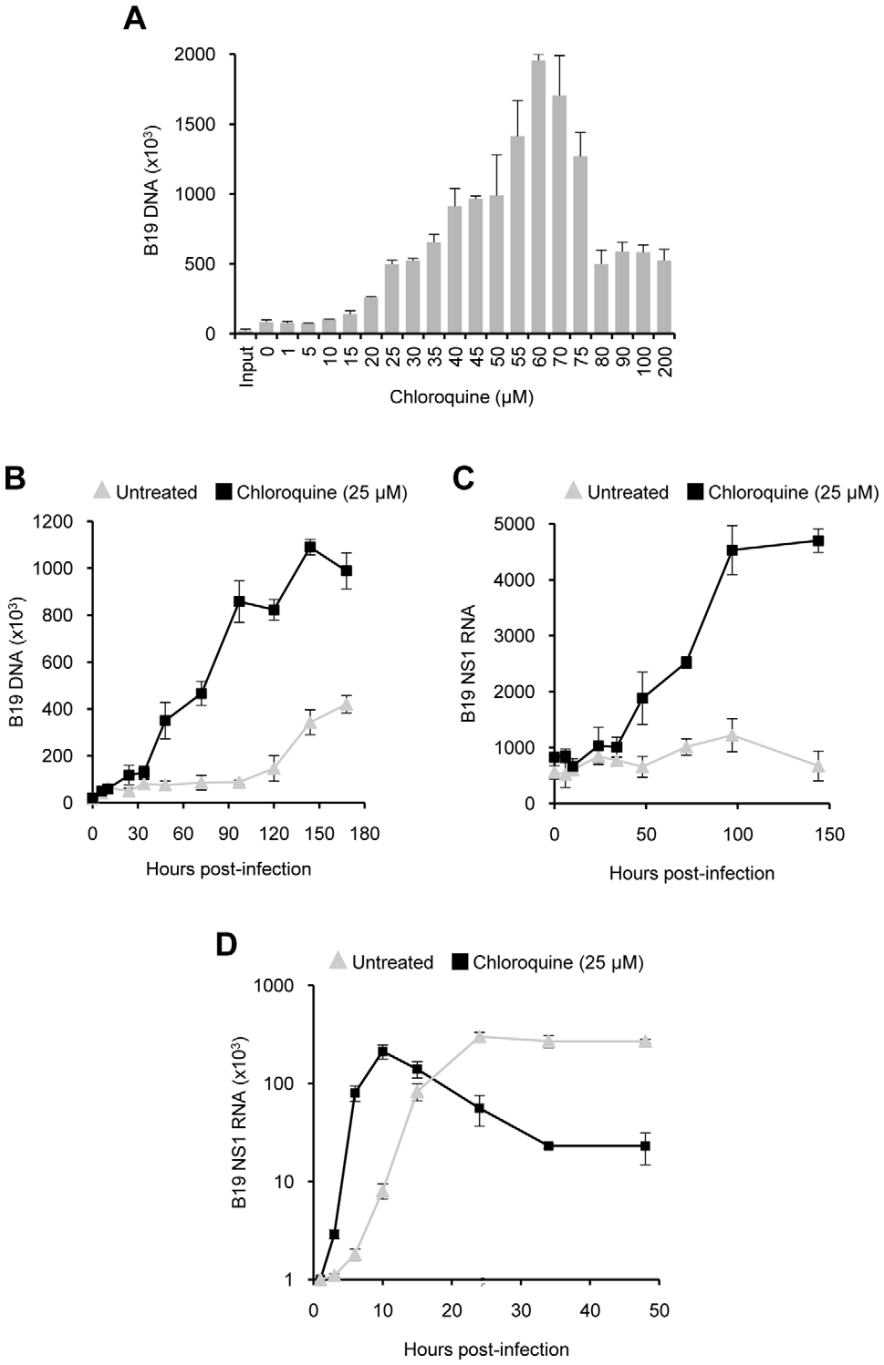
Enhancement of B19V infection by CQ in HepG2 cells and BMMCs. Panel A shows the quantification of viral DNA synthesis from HepG2 cells 90 h after infection with B19V in the presence of increasing CQ concentrations (0–200 µM). The results are the average of three independent experiments. SD bars are shown. Panel B shows the quantification of viral DNA replication at increasing post-infection times in untreated and CQ-treated (25 µM) HepG2 cells. Panel C shows the quantification of viral RNA transcription in HepG2 cells at increasing post-infection times in the presence of 0 or 25 µM CQ. Values shown in panels B and C represent the average of two independent experiments. SD bars are shown. Panel D shows the quantification of viral RNA transcription in BMMCs at increasing post-infection times in the presence of 0 or 25 µM CQ. Results represent mean values from two independent experiments. SD bars are shown.

In the absence of CQ, only a minor amount of viral DNA synthesis was observed starting at 120 h post-infection. No viral RNA could be detected, confirming the poor permissiveness of this cell line for B19V infection. However, in the presence of increasing concentrations of CQ, viral DNA synthesis increased progressively reaching 2,290% at CQ concentrations of 60 µM (Figure 2A). Kinetic studies showed that, in the presence of CQ (25 µM), viral DNA was detected earlier than in untreated cells (Figure 2B). Viral NS1 RNA was only detectable in CQ-treated cells (Figure 2C).

### Effect of CQ on B19V replication in bone marrow mononuclear cells

The presence of CQ (25 µM) accelerated B19V RNA synthesis. However, in CQ-treated cells, viral RNA transcription ceased abruptly and was followed by progressive degradation resembling apoptosis (Figure 2D). Detection of phosphatidyl serine–anexin V complexes by fluorescence microscopy confirmed that the infected BMMCs entered the apoptotic pathway (data not shown). Therefore, the effect of CQ in cultured BMMCs could not be evaluated at stages later that 10 h post-infection. The apoptotic effects were not observed in the cell lines UT7/Epo and HepG2 at concentrations up to 60 µM (data not shown).

### Effect of other antimalarial drugs on B19V viral replication

**Figure 3:**
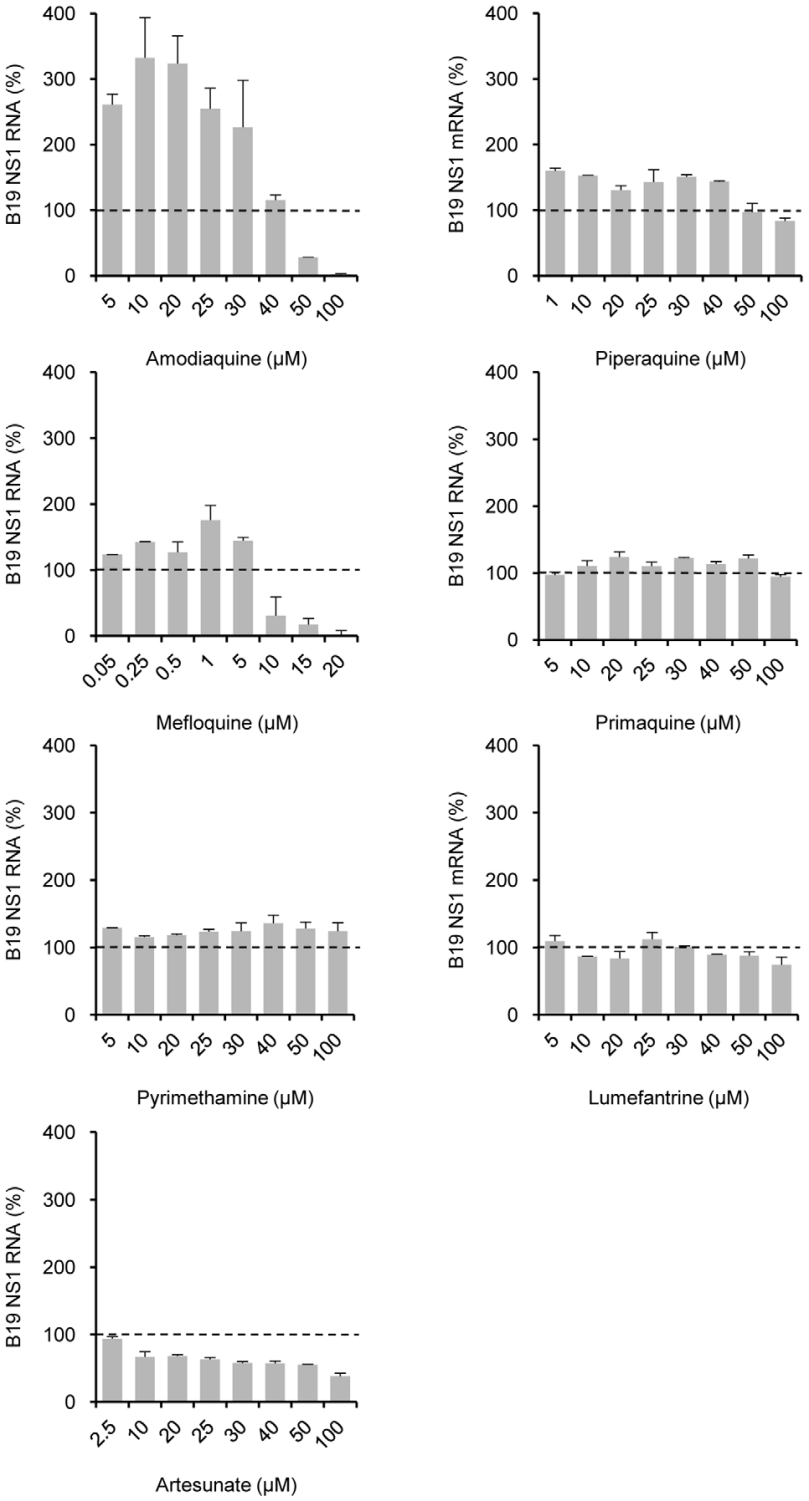
Effect of different antimalarial drugs on B19V infection. UT7/Epo cells were infected with B19V at 4°C for 2 h. The cells were washed with PBS to remove unbound virus and incubated at 37°C in the presence of different drugs. All drugs were used in concentrations ranging from 0 to 100 µM. After 24 h, the amount of B19V NS1 RNA was quantified. The data are expressed as the percentage of the value obtained in untreated cells (dotted line) averaged for two independent experiments. SD bars are shown.

With the exception of the CQ-analogue AQ which enhanced B19V infection at concentrations above 5 µM, no other antimalarial drug had a significant effect on B19V infection (Figure 3). Mild inhibition was observed in the presence of AT, while MQ inhibited the infection at concentrations >10 µM. These effects were also observed when the drugs were added 4 to 7 h post-infection (data not shown), raising the possibility that B19V infectivity was reduced by a drug-specific cytotoxicity.

### CQ prevention of the intracellular degradation of incoming B19V particles

**Figure 4:**
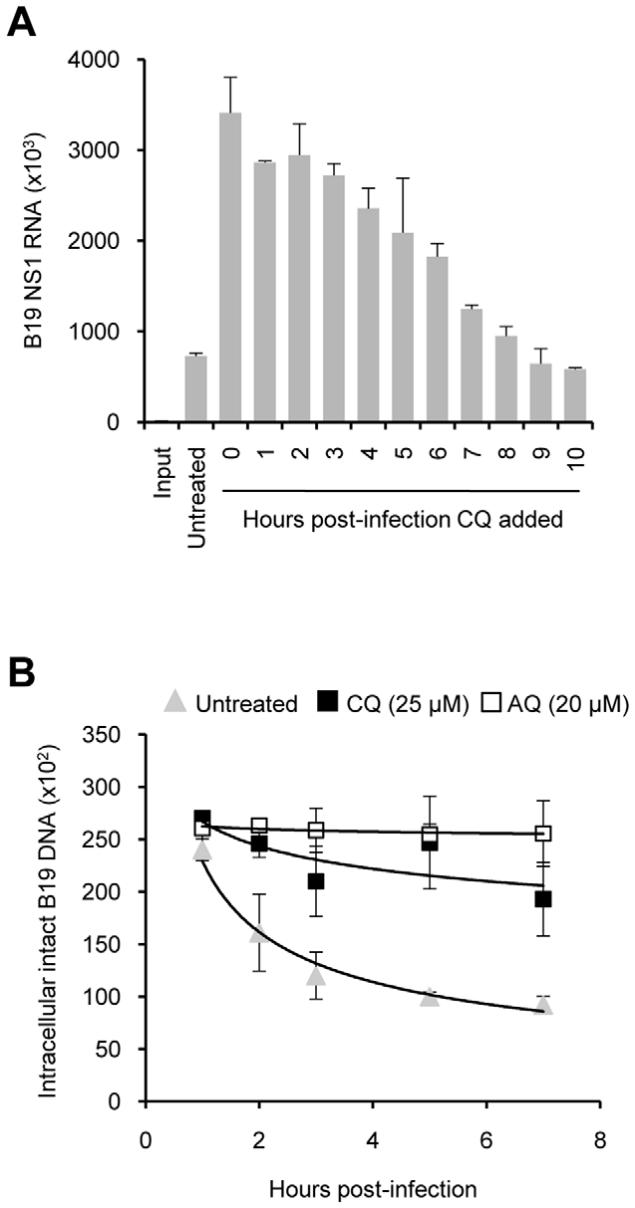
Mechanism of enhancement of B19V infection by 4-aminoquinolines. Panel A shows the decreasing boosting effect of CQ when added at increasing post-infection times. UT7/Epo cells were infected with B19V at 4°C for 2 h. The cells were washed with PBS to remove unbound virus and incubated at 37°C. At progressive post-infection times, CQ (25 µM) was added to the cells. After 24 h post-infection, the viral NS1 RNA was quantified. Results represent mean values from three independent experiments. SD bars are shown. Panel B shows the effect of CQ (25 µM) and AQ (20 µM) on the integrity of the intracellular viral DNA. UT7/Epo cells were infected with B19V. At increasing post-internalization times from 1 to 7 h, the cells were washed and the viral DNA was extracted and quantified. A trendline was plotted using the average values from two independent experiments. SD bars are shown.

The enhancement of B19V infection by CQ decreased progressively with increases in the time at which CQ was added, with no detectable effect at 8–9 h post-infection (Figure 4A). These data indicate that CQ acts early in B19V infection. At progressive times after internalization of B19V, the cells were washed and the viral DNA was quantified. In untreated cells, a progressive degradation of the incoming viral DNA was evident. However, in the presence of CQ (25 µM) or AQ (20 µM), degradation of incoming particles was prevented or minimized (Figure 4B).

### Prevalence and sequelae of B19V infection in PNG children

Serological screening revealed that 3 of 89 healthy control children (3.4%) and 18 of the 111 with severe anemia (16.2%) were IgM positive (*P* = 0.004 by Fisher's exact test). A further 6 B19V IgM-negative children with severe anemia were positive by PCR and 5 of the 18 IgM-positive children were also PCR positive. In 22 of the 24 IgM and/or PCR positive children with severe anemia and plasma available for assay, only 5 did not have detectable 4-aminoquinoline or metabolite concentrations. Based on the respective pharmacokinetic profiles [18], [19], this suggests that most of these children had been treated with either CQ or AQ within the previous 6 weeks.

**Figure 5:**
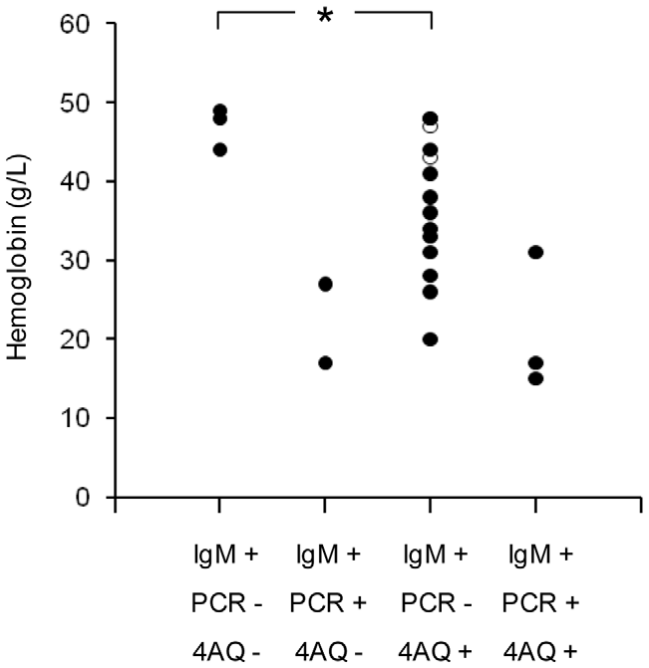
Hemoglobin concentrations in case and control children. Scatter plot showing hemoglobin concentrations in case and control children from a study of severe pediatric infections in PNG. The data are categorised by parvovirus IgM/PCR positivity and prior 4-aminoquinoline (4AQ) use confirmed by drug and metabolite assay. In patients who were either IgM or PCR positive (but not both), 4-aminoquinoline use was associated with a lower hemoglobin (*P = 0.037 by Mann-Whitney U test). The two patients with evidence of treatment with AQ alone are indicated by open circles – all other children received CQ alone or had serum concentrations suggesting recent prior treatment with both CQ and AQ.

Hemoglobin concentrations by B19V IgM/PCR and 4-aminoquinoline status are shown in Figure 5. The lowest concentrations were in the 5 children who were both IgM and PCR positive (i.e. had acute B19 infections). These children had a similar mean age (53 vs 57 months), body weight (15 kg in both groups) and spleen size (5 vs 7 cm) to those children who were either IgM or PCR positive (*P*>0.37 by Mann-Whitney U test) and the percentages with malaria were similar (40.0 vs 44.4%; *P* = 0.63 by Fisher's exact test). In patients who were IgM or PCR positive (indicating a recent but not necessarily acute infection or one which was acute but early in its course), 4-aminoquinoline use was associated with a significantly lower admission hemoglobin concentration (*P* = 0.037). Although the number of patients treated with AQ was small and restricted to children who were IgM positive but PCR negative, they had some of the highest hemoglobin concentrations in this subgroup. This suggests that, consistent with the *in vitro* data, CQ had a greater suppressive effect on bone marrow than AQ in our patients.

## Discussion

Severe anemia is a common and life-threatening complication of malaria in children living in endemic areas [20]. B19V co-infection has been identified as a major factor in its pathogenesis [3], but there are significant regional differences in its seroprevalence and resulting clinical impact [4]–[8] despite the fact that B19V infection is a common childhood illness. Because B19V-associated severe anemia appears to parallel local use of CQ as first-line treatment for malaria, we hypothesized that CQ promotes B19V replication and that, as a consequence, it contributes indirectly to severe anemia. Although a more profound clinical study would be necessary, the present results provide already a strong evidence for this hypothesis.

Apart from its antimalarial effects, CQ has a wide antiviral activity. One of the most important mechanisms of action against viruses is the alkalinization of the endosomal vesicles. In this way, CQ is active against viruses that require a low pH step for cell entry, such as flavivirus, retrovirus and coronavirus [9]. All parvoviruses studied to date also depend on endosomal acidification for cell entry because it facilitates capsid structural transitions [21], [22], and in particular the externalization of the N-terminal region of VP1 which is required for endosomal escape and nuclear targeting [23]. Accordingly, CQ inhibits parvovirus infections. However, we have previously shown that B19V is unique among parvoviruses in that N-VP1 is already externalized on receptor binding [17] and thus not dependent on a low endosomal pH for this critical conformational change. B19V is also unique among parvoviruses for its higher sensitivity to acid degradation [24]. Consequently, CQ-associated alkalinization of endosomal vesicles would be expected to minimize the acidic degradation of incoming B19V particles. Our data confirm that the intracellular degradation of B19V is prevented or minimized in the presence of CQ or AQ. However, other lysosomotropic drugs such as ammonium chloride or bafilomycin A1, which also raise the endosomal pH, had an inhibitory effect on B19V infection in our *in vitro* system (data not shown). Therefore, the mechanism underlying the stabilization of B19V by CQ or AQ is likely to extend beyond pH-neutralizing activity to destabilization of endosome/lysosome membranes typically observed in CQ-treated cells. In this way, CQ would facilitate the endosomal escape of B19V before it reaches the degradative lysosomal compartment and increase the number of particles that can target the nuclei for replication. This is of particular importance since nuclear targeting has been identified as a major limiting factor in parvovirus infections [22], [25].

The plausibility of our *in vitro* observations as an explanation of epidemiological data depends on the pharmacological properties of CQ, especially tissue concentrations. The *in vitro* enhancement of B19V infection by CQ was achieved at concentrations (10–75 µM) that were well above those achieved in plasma after therapeutic doses in children (typically <5 µM) [18]. However, B19V does not replicate in plasma but in tissues, primarily the bone marrow. CQ concentrations in bone marrow are substantially higher than in plasma [26] and have been measured at approximately 100 µM in animal studies [27]. This could reflect, in part, concentration of the drug within precursor cells such as has been observed in circulating erythrocytes [28]. The long terminal elimination half-life of CQ (around 10 days in children) [18] means that conditions favorable to B19V viral replication in bone marrow may persist for several weeks after dosing. In many malaria-endemic regions, antimalarial therapy is given empirically to febrile children without blood smear confirmation. Ironically this might include fever due to B19V itself. The administration of frequent courses of CQ may mean that a child spends long periods of each year at risk of CQ-associated enhanced B19V viremia and its consequences such as anemia.

AQ is a long half-life 4-aminoquinoline compound like CQ and also promoted B19V replication in our *in vitro* experiments. Other drugs tested, including primaquine (an 8-aminoquinoline) and mefloquine (a methanol quinoline), did not influence B19V infection *in vitro*, suggesting that the effect is specific to 4-aminoquinoline compounds. Some other viral infections (SFV, EMCV and EBV) [11], [12] are enhanced by CQ but not by other 4-aminoquinoline antimalarial drugs.

We were able to obtain preliminary human data that are consistent with our laboratory findings. In our 200 unselected PNG children who were participants in a case-control study of severe pediatric infections, we confirmed previous reports that B19V seropositivity is associated with severe anemia and that the lowest hemoglobin concentrations are in those children who had acute infections (i.e. both IgM and PCR positive) [4]. Although there were limited numbers, there was some evidence that prior 4-aminoquinoline, especially CQ, use exacerbates B19V-associated severe anemia apart from in those IgM- and PCR-positive cases who were presumably at the stage of maximal viral replication and consequent bone marrow suppression. Properly designed epidemiological studies in larger, non-convenience samples are, however, needed to confirm these findings.

Although CQ is a safe and inexpensive antimalarial drug, the increasing emergence of resistant *P. falciparum* and *P. vivax* has seen its use decline throughout the tropics. Our data suggest that the prevalence of severe malarial anemia should also fall as a result. However, when an effective B19V vaccine becomes available, this should be considered a priority intervention where pediatric B19V seroprevalence rates are high and other causes of anemia such as nutritional deficiency and intestinal parasitic infection are present. Artemisinin-based combination therapy (ACT) is the current WHO-recommended first-line treatment for uncomplicated malaria [29]. Our data suggest that, pending more definitive *in vivo* data including appropriately designed clinical trials, a non-4-aminoquinoline drug should be preferred to partner the artemisinin derivative so that the contribution of B19V to severe anemia is minimized.
